# Palmoplantar Pustulosis: A Case Report

**DOI:** 10.5811/cpcem.2020.7.48476

**Published:** 2020-09-17

**Authors:** Anne Dulski, Vince Varamo

**Affiliations:** Kent Hospital, Department of Emergency Medicine, Warwick, Rhode Island

**Keywords:** Palmoplantar pustulosis, rash, emergency medicine

## Abstract

**Introduction:**

Dermatology complaints account for 3.3% of emergency department (ED) visits per year. Most rashes are benign, but there are a select few that emergency physicians must be familiar with as delay in treatment could be life threatening.

**Case Report:**

A well-appearing, 76-year-old male presented to the ED with multiple coalescing pustules to his palms and soles and was transferred to the nearest tertiary care hospital for dermatology consult. He was diagnosed with palmoplantar pustulosis and discharged home with a five-day course of clobetasol propionate 0.05% cream twice daily and outpatient dermatology follow-up.

**Conclusion:**

Palmoplantar pustulosis is an uncommon skin condition characterized by recurrent eruptions of sterile pustules localized to the palms and soles. Emergency physician awareness of this rare diagnosis may help prevent hospital admissions and lead to earlier initiation of treatment with outpatient dermatology follow-up.

## INTRODUCTION

The chief complaint of rash is a common reason patients present to the emergency department (ED). It is estimated that dermatology complaints account for 3.3% of all ED visits per year.^1^ Although patients who present to the ED with rashes are usually well appearing and can be treated with outpatient management, there are a select few conditions that emergency physicians must be aware of as proper diagnosis and treatment could prevent further complications and even death. Palmoplantar pustulosis (PPP) is an uncommon, chronic skin condition characterized by recurrent eruptions of sterile pustules localized to the palms and soles.^2^ Because it is an uncommon diagnosis data pertaining to it is limited. It is thought that PPP usually develops in middle-aged adults, 50–69, and occurs more in females.^3^ Classical findings of PPP include eruptions of sterile pustules on the palms and/or soles with associated scaling, erythema, pruritus, burning, and/or pain.^4^,^5^ The diagnosis is based primarily on history and physical.

First-line treatment includes topical corticosteroids and smoking cessation; more advanced therapy includes oral retinoids, photochemotherapy, immunosuppressants and, if there is no improvement, anti-tumor necrosis factor drugs.^6^,^7^ The patient we describe presented with an acute rash to the palms and soles consistent with this disease with no history of psoriasis or skin disorders. Due to the rarity of this disease and broad differential at presentation, the patient was transferred to a nearby tertiary care center with dermatology referral. The more that is known about PPP, the sooner patients can be diagnosed correctly and started on the appropriate course of treatment.

## CASE REPORT

**Image 1:**
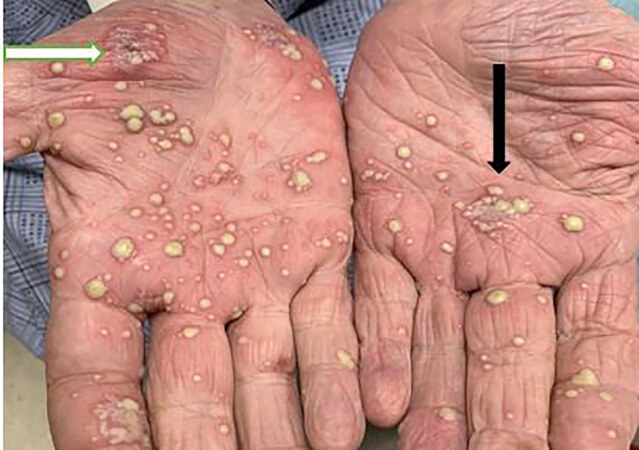
Patient’s palms with black arrow pointing to coalescing pustules with surrounding erythema and white arrow pointing to pustules with scaling consistent with palmoplantar pustulosis.

**Image 2:**
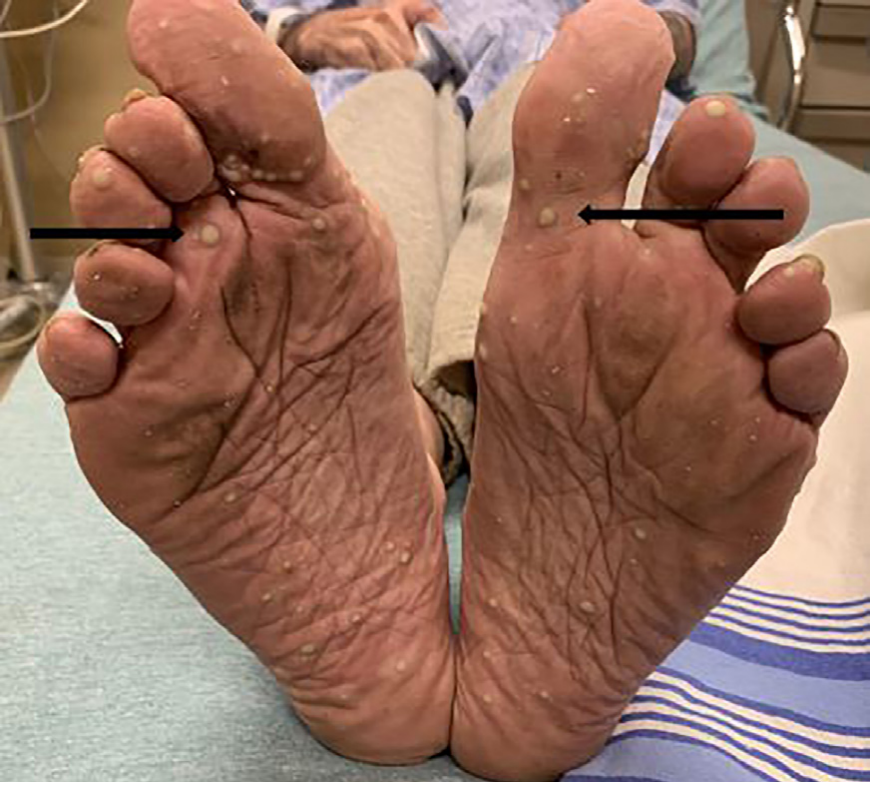
Patient’s feet with black arrows pointing to pustules on the soles consistent with palmoplantar pustulosis.

**Image 3:**
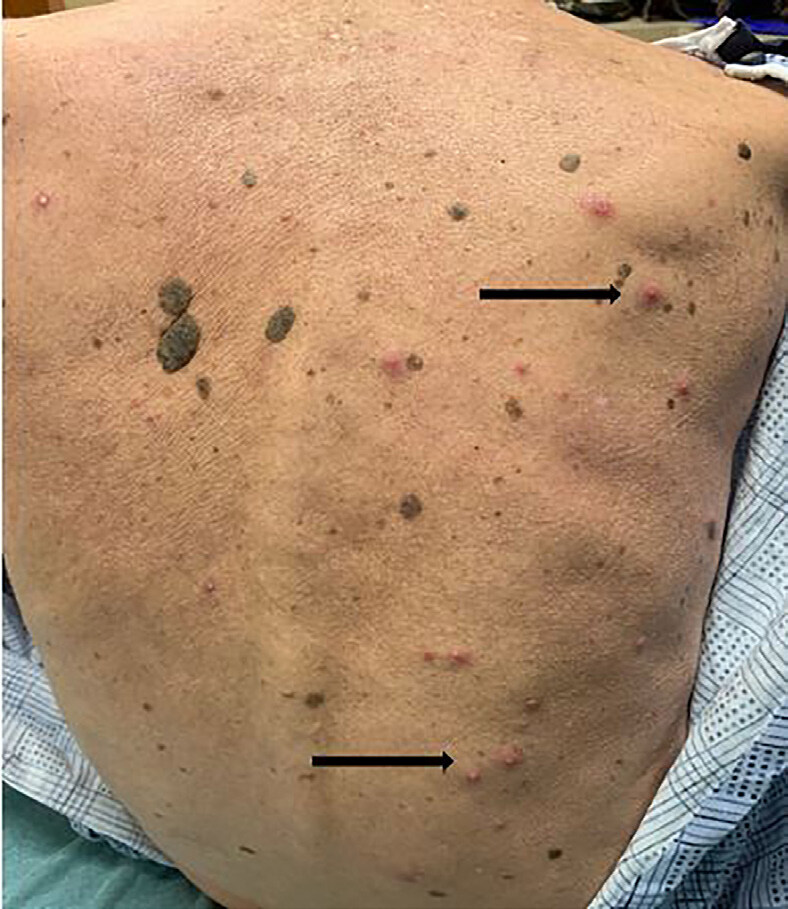
Scattered pustules with surrounding erythema on the patient’s back as indicated by the black arrows.

A 76-year-old male, daily smoker, with a history of chronic obstructive lung disease, peptic ulcer disease, prostate cancer status post prostatectomy in 1996, presented to the ED with a new rash to his palms. The rash started four to five days prior and had been progressively worsening without pain or pruritus. He admitted to using a furniture polish without gloves the day prior to the rash starting but had used it in the past without any side effects. He denied any systemic symptoms, recent illnesses, new or current medications, or sexual activity. On initial presentation, the patient’s blood pressure was 119/66 millimeters of mercury, heart rate 76 beats per minutes, temperature 36.7º° Celsius and his oxygen saturation was 99% on room air. Physical exam revealed multiple, coalescing pustules on background erythema on the palms and soles (Images 1 and 2) with a few diffusely scattered pustules to his back (Image 3) and abdomen. There was no mucosal involvement.

Workup in the ED included a complete blood count, comprehensive metabolic panel, C-reactive protein, erythrocyte sediment rate, herpes simplex virus, hepatitis panel, gonorrhea, chlamydia, and syphilis testing. Due to all labs being within normal limits, the decision was made to transfer to a nearby tertiary care center for urgent dermatology referral. During his admission, he was evaluated by dermatology and hematology. Lab work showed a 20.6% monocytosis (normal monocyte range 2–8%) but was otherwise unremarkable, and a potassium hydroxide prep was negative for fungal infection. Skin biopsy results showed acute spongiolitic dermatitis with subcorneal pustules composed of neutrophils, consistent with pustular psoriasis. He was diagnosed with PPP and discharged home after five days with clobetasol propionate 0.05% cream twice daily and outpatient dermatology follow-up.

CPC-EM Capsule

What do we already know about this clinical entity?

*Palmoplantar pustulosis is characterized by sterile pustules to the palms and/or soles; treatment includes clobetasol propionate cream and smoking cessation*.

What makes this presentation of disease reportable?

*The patient had no history of skin conditions and had an acute eruption of a rash that is not commonly seen in the emergency department*.

What is the major learning point?

*Palmoplantar pustulosis is a rare but benign skin condition that emergency physicians should be aware of to aid in early diagnosis and initiation of treatment*.

How might this improve emergency medicine practice?

*Awareness of this rare diagnosis may help prevent hospital admissions and lead to earlier initiation of treatment*.

## DISCUSSION

Palmoplantar pustulosis is an uncommon skin disorder with a presumed prevalence of less than 1% of the population.^2^ It is characterized by recurrent eruptions of sterile pustules primarily localized to the palms and/or soles.^2^ Some consider PPP to be a subtype of psoriasis, but others suggest it is a separate entity.^5^ It is thought that PPP usually develops in middle-aged adults, 50–69, and occurs more often in females.^3^ While the pathogenesis is unknown, studies have suggested an inflammatory process that destroys the acrosyringium (intraepidermal eccrine sweat ducts) and a possible association with increased interleukin-8, interleukin-17, tumor necrosis factor-alpha, interleukin-22, and interferon-gamma.^8^ Proposed environmental factors that may contribute to the onset of PPP include smoking, stress, infection, genetics, and cessation or initiation of certain medications.^4^,^9^ The associated symptoms of scaling dry skin can lead to painful cracks and fissures that can make activities of daily living challenging and have a negative impact on a patient’s life.^10^

Diagnosis is based on history and physical examination because lab work is usually unremarkable. Skin biopsies are often not necessary but may help in supporting the diagnosis if there is uncertainty.^2^,^5^ The recommended first-line treatment for PPP is high potency clobetasol propionate 0.05% cream twice daily for at least four weeks.^9^,^11^ In addition to a steroid cream, first-line treatment options include daily living changes. Research has found a strong association between PPP and smoking; and patients who successfully quit have been found to have a decrease in their symptoms and recurrence rate.^4^ In addition to smoking cessation, other behavioral changes include daily skin moisturizing and avoidance of skin irritants. Due to the rarity of this skin condition, there remains incomplete consensus of the best tr eatment; however, topical steroids have been found to be the most effective and have the least amount of side effects. Other treatm ent options include oral retinoids and photochemotherapy, but side effects of both limit their use for second-line therapy.^12^,^13^ Palmoplantar pustulosis is a chronic recurrent skin condition and many patients will need to be on lifelong therapy for symptomatic relief and to decrease recurrence rate.^14^

## CONCLUSION

Palmoplantar pustulosis is an uncommon skin disorder that is characterized by recurrent eruptions of sterile pustules primarily localized to the palms and/or soles. Emergency physician awareness of this rare diagnosis may help prevent hospital admissions and lead to earlier initiation of treatment with outpatient dermatology follow-up.
